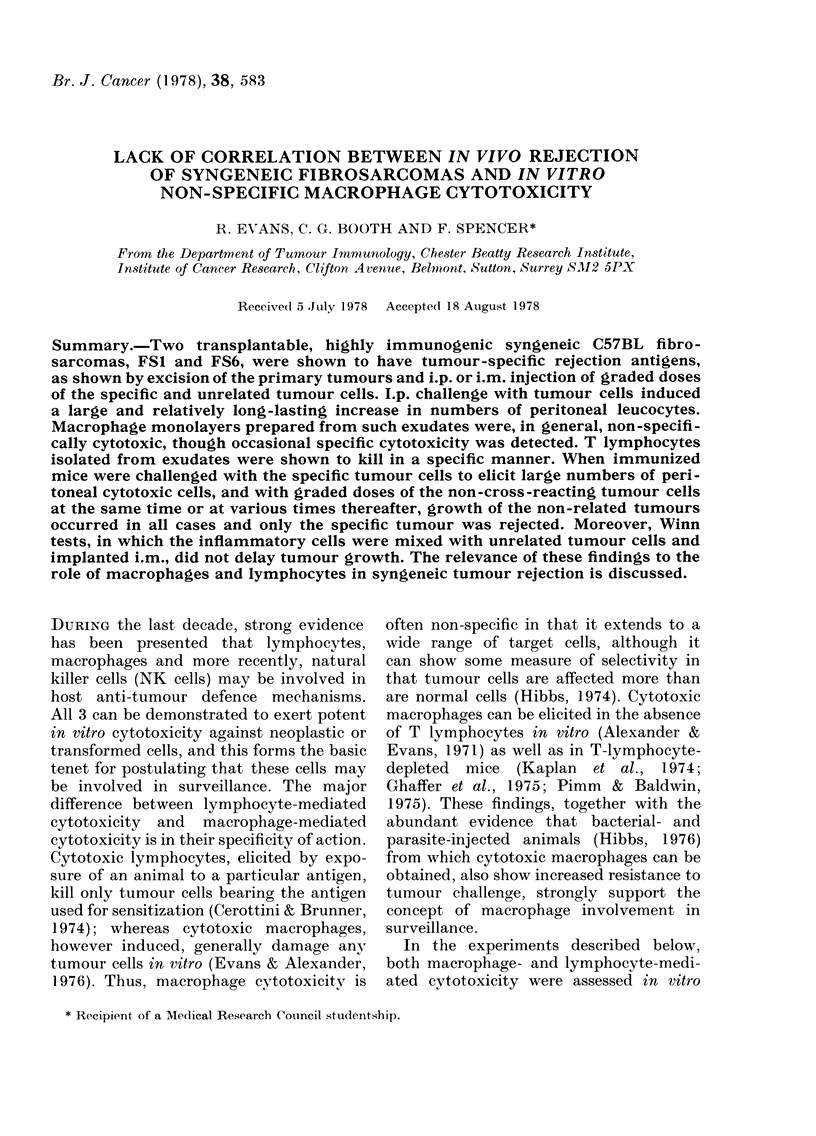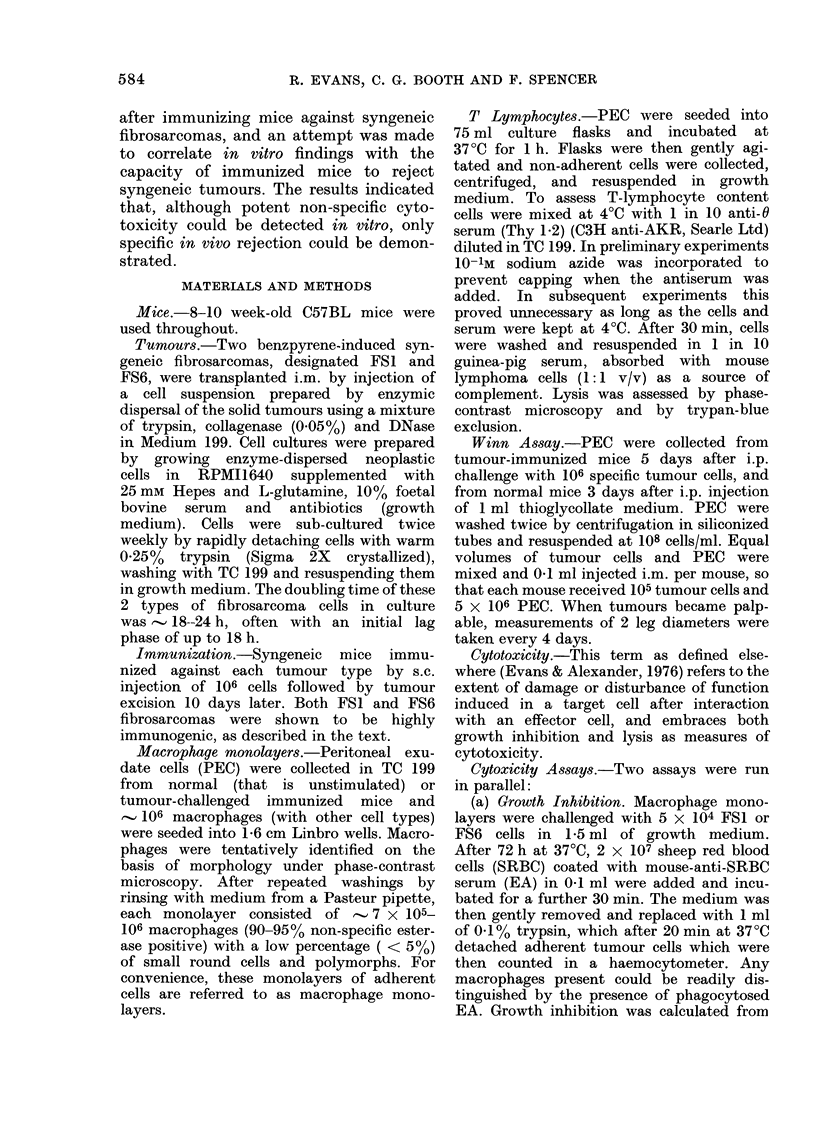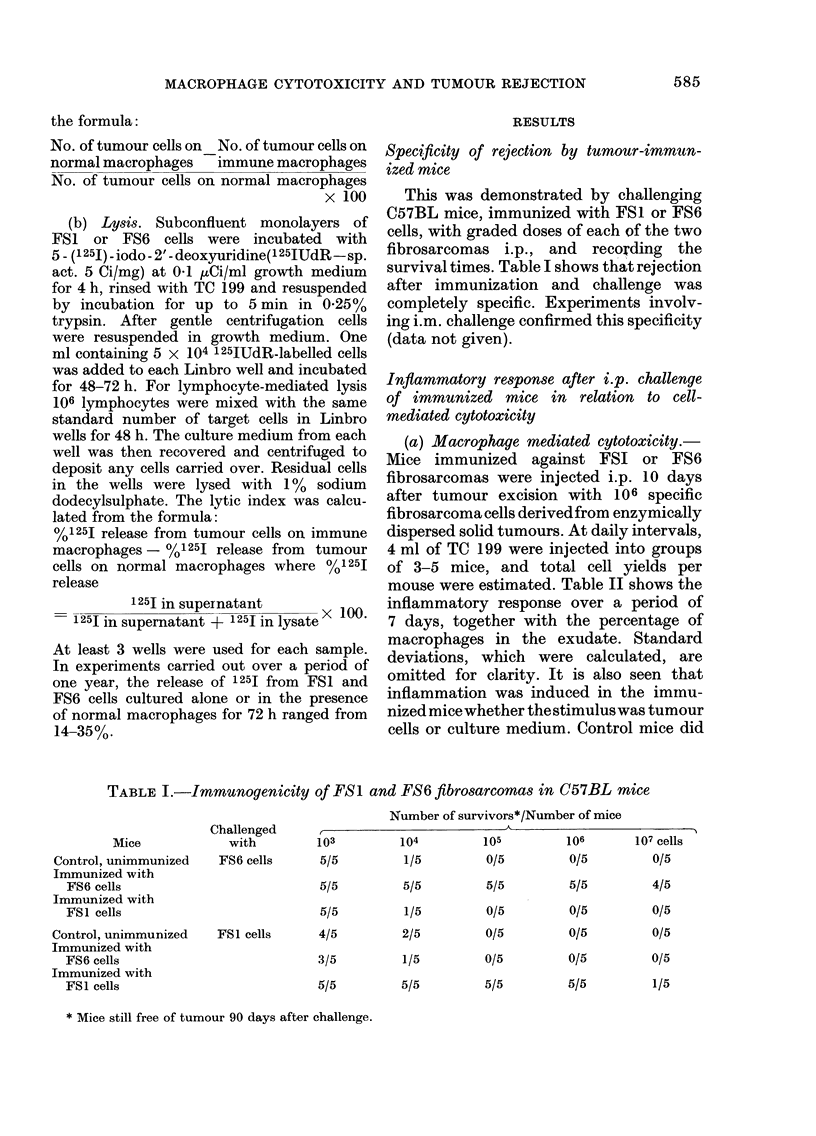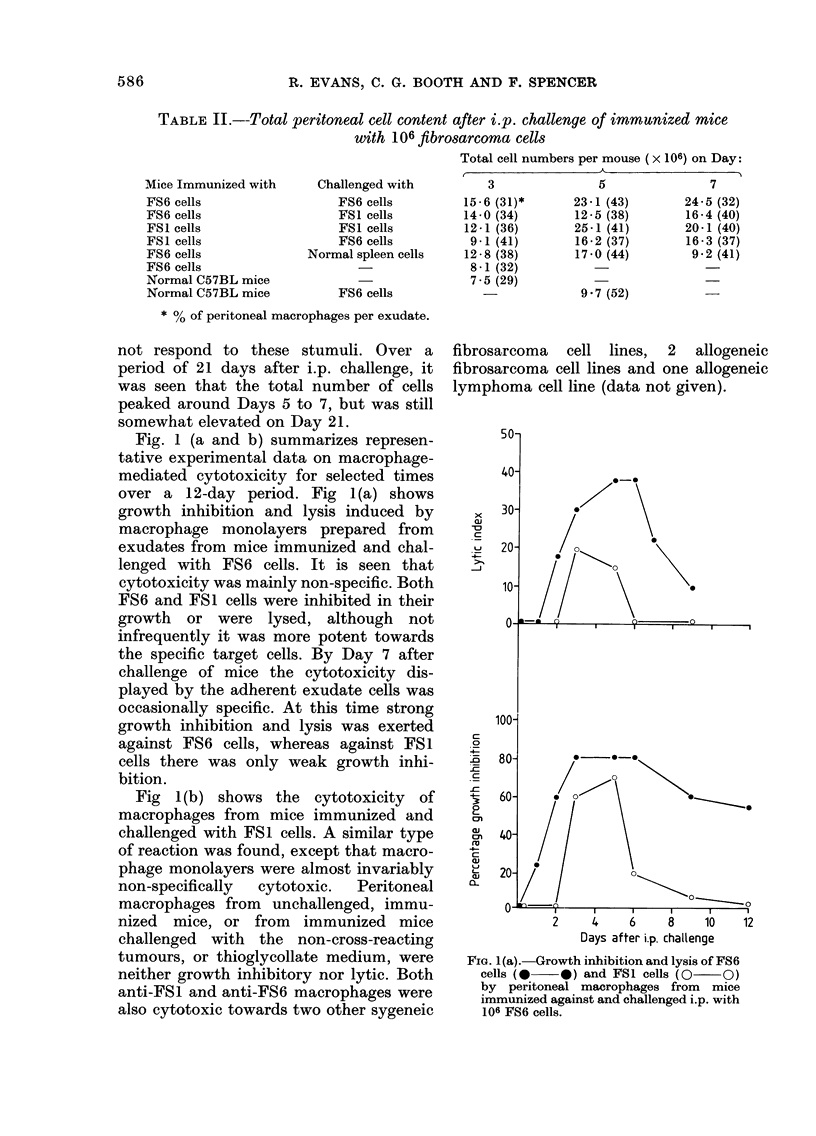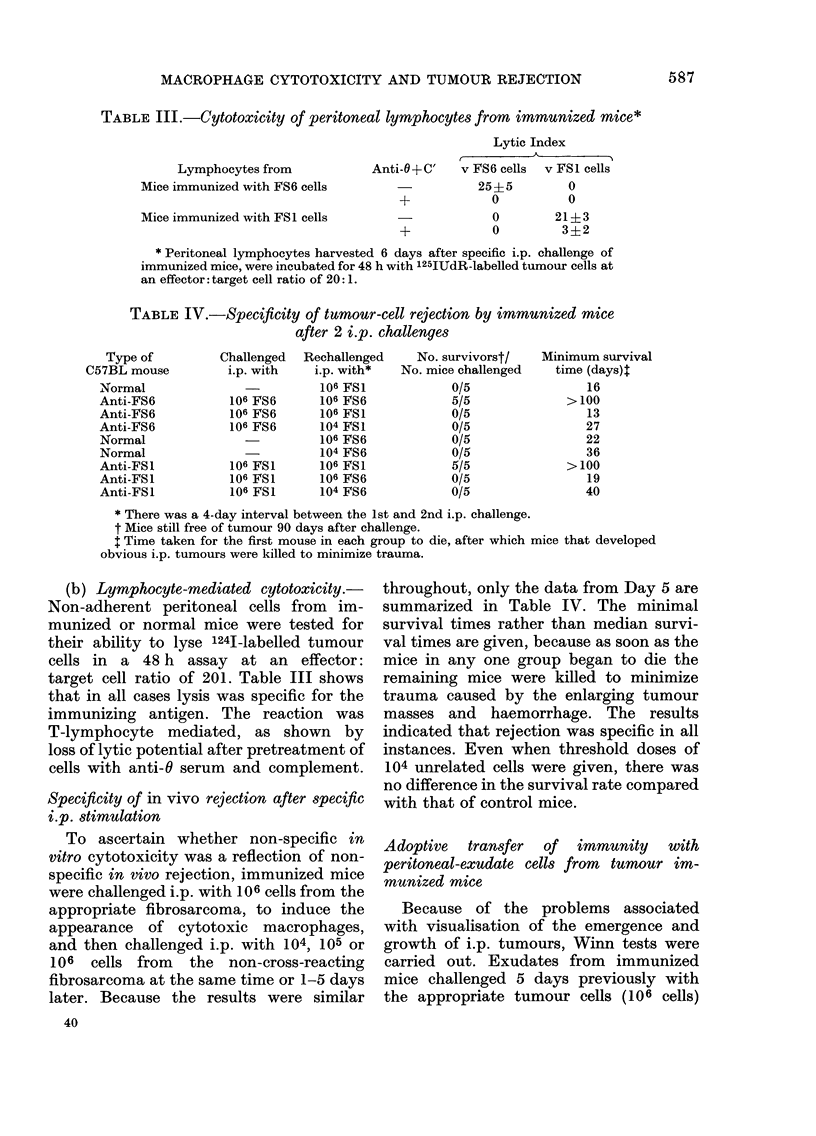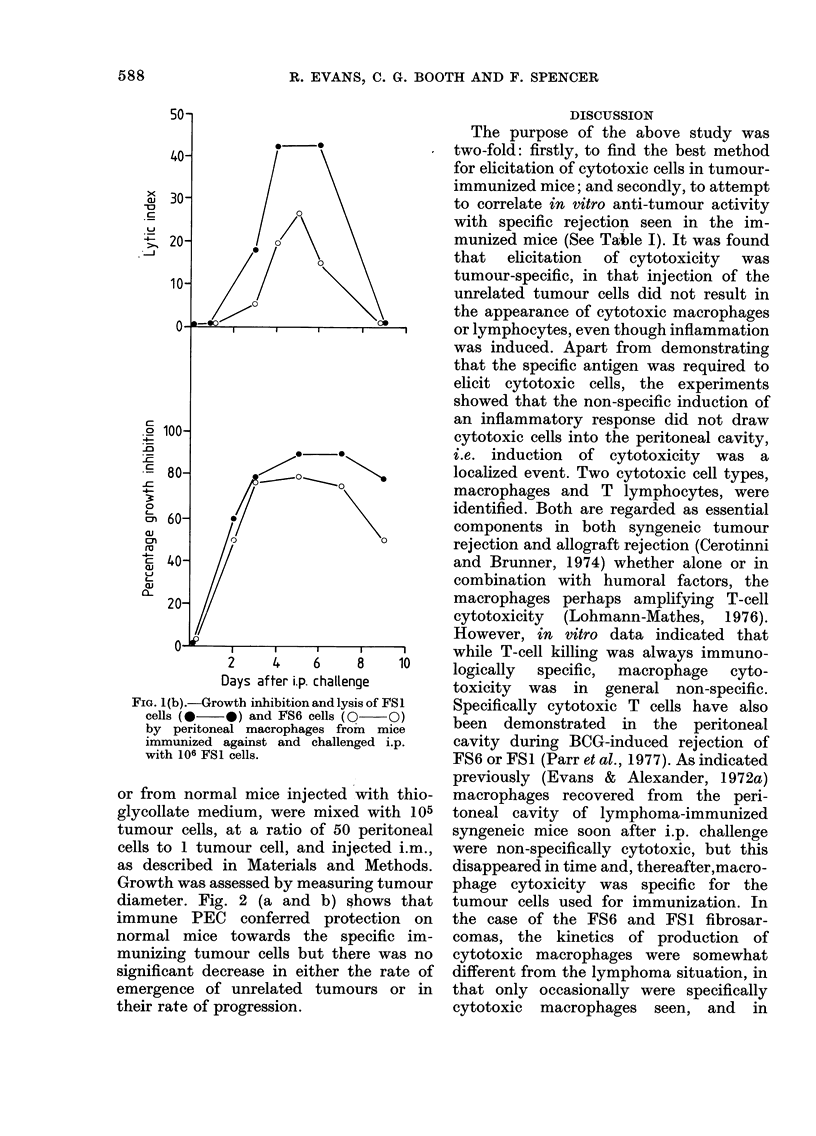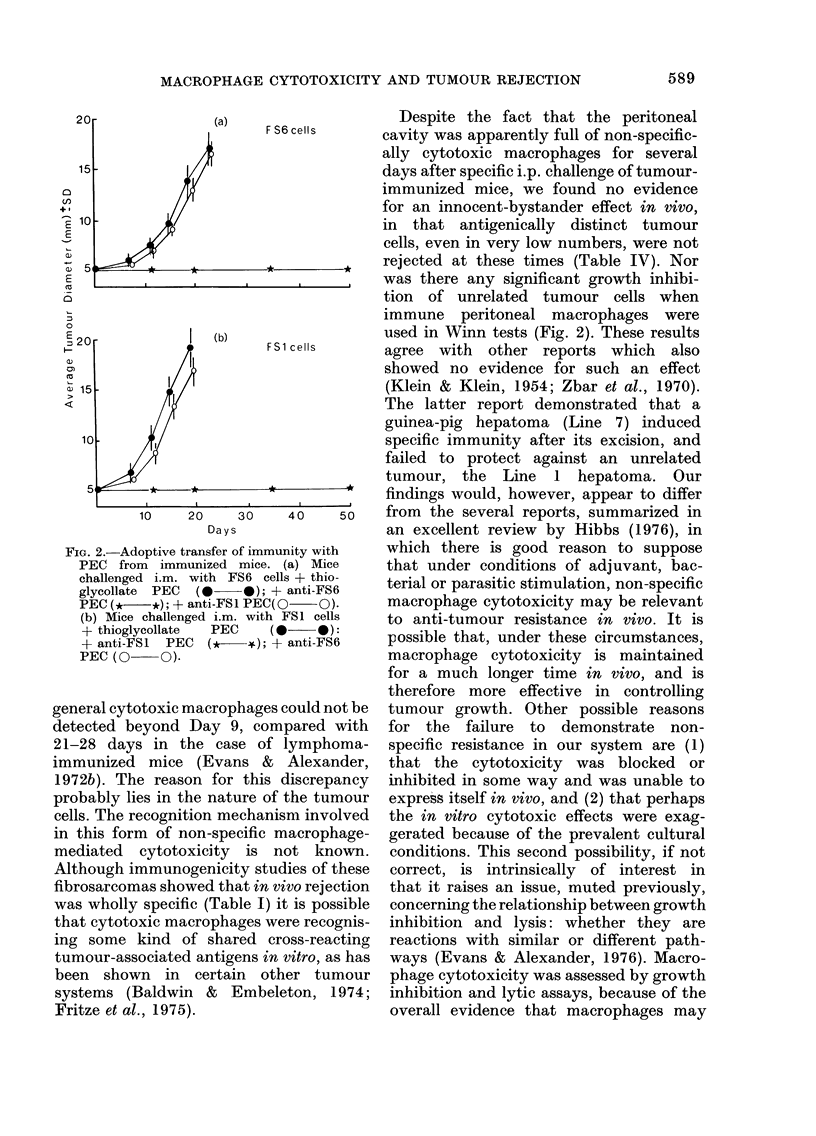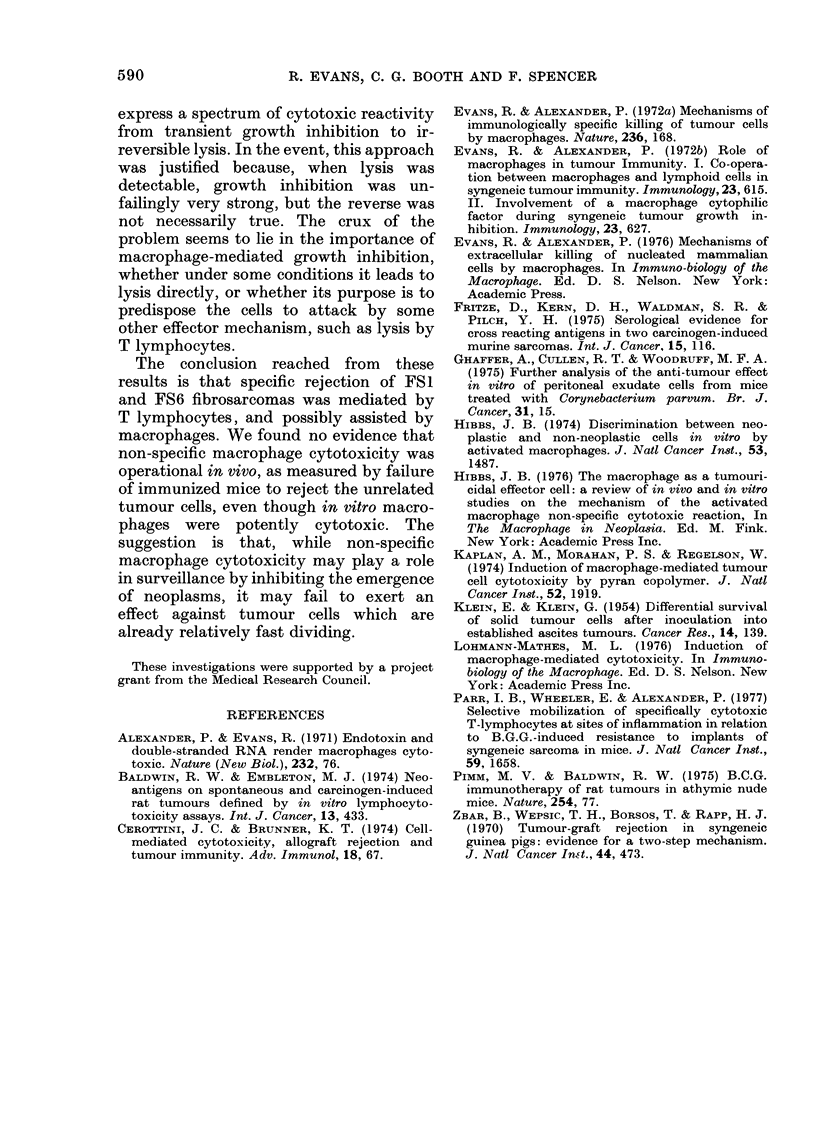# Lack of correlation between in vivo rejection of syngeneic fibrosarcomas and in vitro non-specific macrophage cytotoxicity.

**DOI:** 10.1038/bjc.1978.253

**Published:** 1978-11

**Authors:** R. Evans, C. G. Booth, F. Spencer

## Abstract

Two transplantable, highly immunogenic syngeneic C57BL fibrosarcomas, FS1 and FS6, were shown to have tumour-specific rejection antigens, as shown by excision of the primary tumours and i.p. or i.m. injection of graded doses of the specific and unrelated tumour cells. I.p. challenge with tumour cells induced a large and relatively long-lasting increase in numbers of peritoneal leucocytes. Macrophage monolayers prepared from such exudates were, in general, non-specifically cytotoxic, though occasional specific cytotoxicity was detected. T lymphocytes isolated from exudates were shown to kill in a specific manner. When immunized mice were challenged with the specific tumour cells to elicit large numbers of peritoneal cytotoxic cells, and with graded doses of the non-cross-reacting tumour cells at the same time or at various times thereafter, growth of the non-related tumours occurred in all cases and only the specific tumour was rejected. Moreover, Winn tests, in which the inflammatory cells were mixed with unrelated tumour cells and implanted i.m., did not delay tumour growth. The relevance of these findings to the role of macrophages and lymphocytes in syngeneic tumour rejection is discussed.


*Br. J. Cancer* (1978), **38**, 583

### LACK OF CORRELATION BETWEEN *IN VIVO* REJECTION OF SYNGENEIC FIBROSARCOMAS AND *IN VITRO* NON-SPECIFIC MACROPHAGE CYTOTOXICITY

R. EVANS, C. G. BOOTH AND F. SPENCER*

*From the Department of Tumour Immunology, Chester Beatty Research Institute, Institute of Cancer Research, Clifton Avenue, Belmont, Sutton, Surrey SM2 5PX*

Received 5 July 1978 Accepted 18 August 1978

**Summary.—Two transplantable, highly immunogenic syngeneic C57BL fibrosarcomas, FS1 and FS6, were shown to have tumour-specific rejection antigens, as shown by excision of the primary tumours and i.p. or i.m. injection of graded doses of the specific and unrelated tumour cells. I.p. challenge with tumour cells induced a large and relatively long-lasting increase in numbers of peritoneal leucocytes. Macrophage monolayers prepared from such exudates were, in general, non-specifically cytotoxic, though occasional specific cytotoxicity was detected. T lymphocytes isolated from exudates were shown to kill in a specific manner. When immunized mice were challenged with the specific tumour cells to elicit large numbers of peritoneal cytotoxic cells, and with graded doses of the non-cross-reacting tumour cells at the same time or at various times thereafter, growth of the non-related tumours occurred in all cases and only the specific tumour was rejected. Moreover, Winn tests, in which the inflammatory cells were mixed with unrelated tumour cells and implanted i.m., did not delay tumour growth. The relevance of these findings to the role of macrophages and lymphocytes in syngeneic tumour rejection is discussed.**

DURING the last decade, strong evidence has been presented that lymphocytes, macrophages and more recently, natural killer cells (NK cells) may be involved in host anti-tumour defence mechanisms. All 3 can be demonstrated to exert potent *in vitro* cytotoxicity against neoplastic or transformed cells, and this forms the basic tenet for postulating that these cells may be involved in surveillance. The major difference between lymphocyte-mediated cytotoxicity and macrophage-mediated cytotoxicity is in their specificity of action. Cytotoxic lymphocytes, elicited by exposure of an animal to a particular antigen, kill only tumour cells bearing the antigen used for sensitization (Cerottini & Brunner, 1974); whereas cytotoxic macrophages, however induced, generally damage any tumour cells *in vitro* (Evans & Alexander, 1976). Thus, macrophage cytotoxicity is often non-specific in that it extends to a wide range of target cells, although it can show some measure of selectivity in that tumour cells are affected more than are normal cells (Hibbs, 1974). Cytotoxic macrophages can be elicited in the absence of T lymphocytes *in vitro* (Alexander & Evans, 1971) as well as in T-lymphocyte-depleted mice (Kaplan *et al.*, 1974; Ghaffer *et al.*, 1975; Pimm & Baldwin, 1975). These findings, together with the abundant evidence that bacterial- and parasite-injected animals (Hibbs, 1976) from which cytotoxic macrophages can be obtained, also show increased resistance to tumour challenge, strongly support the concept of macrophage involvement in surveillance.

In the experiments described below, both macrophage- and lymphocyte-mediated cytotoxicity were assessed *in vitro*

* Recipient of a Medical Research Council studentship.

after immunizing mice against syngeneic fibrosarcomas, and an attempt was made to correlate *in vitro* findings with the capacity of immunized mice to reject syngeneic tumours. The results indicated that, although potent non-specific cytotoxicity could be detected *in vitro*, only specific *in vivo* rejection could be demonstrated.

### MATERIALS AND METHODS

*Mice.*—8–10 week-old C57BL mice were used throughout.

*Tumours.*—Two benzpyrene-induced syngeneic fibrosarcomas, designated FS1 and FS6, were transplanted i.m. by injection of a cell suspension prepared by enzymic dispersal of the solid tumours using a mixture of trypsin, collagenase (0·05%) and DNase in Medium 199. Cell cultures were prepared by growing enzyme-dispersed neoplastic cells in RPMI1640 supplemented with 25 mM Hepes and L-glutamine, 10% foetal bovine serum and antibiotics (growth medium). Cells were sub-cultured twice weekly by rapidly detaching cells with warm 0·25% trypsin (Sigma 2X crystallized), washing with TC 199 and resuspending them in growth medium. The doubling time of these 2 types of fibrosarcoma cells in culture was ~ 18–24 h, often with an initial lag phase of up to 18 h.

*Immunization.*—Syngeneic mice immunized against each tumour type by s.c. injection of $10^6$ cells followed by tumour excision 10 days later. Both FS1 and FS6 fibrosarcomas were shown to be highly immunogenic, as described in the text.

*Macrophage monolayers.*—Peritoneal exudate cells (PEC) were collected in TC 199 from normal (that is unstimulated) or tumour-challenged immunized mice and ~ $10^6$ macrophages (with other cell types) were seeded into 1·6 cm Linbro wells. Macrophages were tentatively identified on the basis of morphology under phase-contrast microscopy. After repeated washings by rinsing with medium from a Pasteur pipette, each monolayer consisted of ~ $7 \times 10^5$–$10^6$ macrophages (90–95% non-specific esterase positive) with a low percentage ( < 5%) of small round cells and polymorphs. For convenience, these monolayers of adherent cells are referred to as macrophage monolayers.

*T Lymphocytes.*—PEC were seeded into 75 ml culture flasks and incubated at 37°C for 1 h. Flasks were then gently agitated and non-adherent cells were collected, centrifuged, and resuspended in growth medium. To assess T-lymphocyte content cells were mixed at 4°C with 1 in 10 anti-$\theta$ serum (Thy 1·2) (C3H anti-AKR, Searle Ltd) diluted in TC 199. In preliminary experiments $10^{-1}$M sodium azide was incorporated to prevent capping when the antiserum was added. In subsequent experiments this proved unnecessary as long as the cells and serum were kept at 4°C. After 30 min, cells were washed and resuspended in 1 in 10 guinea-pig serum, absorbed with mouse lymphoma cells (1:1 v/v) as a source of complement. Lysis was assessed by phase-contrast microscopy and by trypan-blue exclusion.

*Winn Assay.*—PEC were collected from tumour-immunized mice 5 days after i.p. challenge with $10^6$ specific tumour cells, and from normal mice 3 days after i.p. injection of 1 ml thioglycollate medium. PEC were washed twice by centrifugation in siliconized tubes and resuspended at $10^8$ cells/ml. Equal volumes of tumour cells and PEC were mixed and 0·1 ml injected i.m. per mouse, so that each mouse received $10^5$ tumour cells and $5 \times 10^6$ PEC. When tumours became palpable, measurements of 2 leg diameters were taken every 4 days.

*Cytotoxicity.*—This term as defined elsewhere (Evans & Alexander, 1976) refers to the extent of damage or disturbance of function induced in a target cell after interaction with an effector cell, and embraces both growth inhibition and lysis as measures of cytotoxicity.

*Cytoxicity Assays.*—Two assays were run in parallel:

(a) *Growth Inhibition.* Macrophage monolayers were challenged with $5 \times 10^4$ FS1 or FS6 cells in 1·5 ml of growth medium. After 72 h at 37°C, $2 \times 10^7$ sheep red blood cells (SRBC) coated with mouse-anti-SRBC serum (EA) in 0·1 ml were added and incubated for a further 30 min. The medium was then gently removed and replaced with 1 ml of 0·1% trypsin, which after 20 min at 37°C detached adherent tumour cells which were then counted in a haemocytometer. Any macrophages present could be readily distinguished by the presence of phagocytosed EA. Growth inhibition was calculated from the formula:

$$\frac{\text{No. of tumour cells on normal macrophages} - \text{No. of tumour cells on immune macrophages}}{\text{No. of tumour cells on normal macrophages}} \times 100$$

(b) *Lysis*. Subconfluent monolayers of FS1 or FS6 cells were incubated with 5-($^{125}$I)-iodo-2′-deoxyuridine($^{125}$IUdR—sp. act. 5 Ci/mg) at 0·1 $\mu$Ci/ml growth medium for 4 h, rinsed with TC 199 and resuspended by incubation for up to 5 min in 0·25% trypsin. After gentle centrifugation cells were resuspended in growth medium. One ml containing $5 \times 10^4$ $^{125}$IUdR-labelled cells was added to each Linbro well and incubated for 48–72 h. For lymphocyte-mediated lysis $10^6$ lymphocytes were mixed with the same standard number of target cells in Linbro wells for 48 h. The culture medium from each well was then recovered and centrifuged to deposit any cells carried over. Residual cells in the wells were lysed with 1% sodium dodecylsulphate. The lytic index was calculated from the formula:
%$^{125}$I release from tumour cells on immune macrophages — %$^{125}$I release from tumour cells on normal macrophages where %$^{125}$I release

$$= \frac{^{125}\text{I in supernatant}}{^{125}\text{I in supernatant} + {}^{125}\text{I in lysate}} \times 100.$$

At least 3 wells were used for each sample. In experiments carried out over a period of one year, the release of $^{125}$I from FS1 and FS6 cells cultured alone or in the presence of normal macrophages for 72 h ranged from 14–35%.

### RESULTS

*Specificity of rejection by tumour-immunized mice*

This was demonstrated by challenging C57BL mice, immunized with FS1 or FS6 cells, with graded doses of each of the two fibrosarcomas i.p., and recording the survival times. Table I shows that rejection after immunization and challenge was completely specific. Experiments involving i.m. challenge confirmed this specificity (data not given).

*Inflammatory response after i.p. challenge of immunized mice in relation to cell-mediated cytotoxicity*

(*a*) *Macrophage mediated cytotoxicity.*—Mice immunized against FSI or FS6 fibrosarcomas were injected i.p. 10 days after tumour excision with $10^6$ specific fibrosarcoma cells derived from enzymically dispersed solid tumours. At daily intervals, 4 ml of TC 199 were injected into groups of 3–5 mice, and total cell yields per mouse were estimated. Table II shows the inflammatory response over a period of 7 days, together with the percentage of macrophages in the exudate. Standard deviations, which were calculated, are omitted for clarity. It is also seen that inflammation was induced in the immunized mice whether the stimulus was tumour cells or culture medium. Control mice did

TABLE I.—*Immunogenicity of FS*1 *and FS*6 *fibrosarcomas in C*57*BL mice*

| Mice | Challenged with | Number of survivors*/Number of mice | | | | |
|---|---|---|---|---|---|---|
| | | $10^3$ | $10^4$ | $10^5$ | $10^6$ | $10^7$ cells |
| Control, unimmunized | FS6 cells | 5/5 | 1/5 | 0/5 | 0/5 | 0/5 |
| Immunized with FS6 cells | | 5/5 | 5/5 | 5/5 | 5/5 | 4/5 |
| Immunized with FS1 cells | | 5/5 | 1/5 | 0/5 | 0/5 | 0/5 |
| Control, unimmunized | FS1 cells | 4/5 | 2/5 | 0/5 | 0/5 | 0/5 |
| Immunized with FS6 cells | | 3/5 | 1/5 | 0/5 | 0/5 | 0/5 |
| Immunized with FS1 cells | | 5/5 | 5/5 | 5/5 | 5/5 | 1/5 |

* Mice still free of tumour 90 days after challenge.

TABLE II.—*Total peritoneal cell content after i.p. challenge of immunized mice with* $10^6$ *fibrosarcoma cells*

| Mice Immunized with | Challenged with | Total cell numbers per mouse ($\times 10^6$) on Day: 3 | 5 | 7 |
|---|---|---|---|---|
| FS6 cells | FS6 cells | 15·6 (31)* | 23·1 (43) | 24·5 (32) |
| FS6 cells | FS1 cells | 14·0 (34) | 12·5 (38) | 16·4 (40) |
| FS1 cells | FS1 cells | 12·1 (36) | 25·1 (41) | 20·1 (40) |
| FS1 cells | FS6 cells | 9·1 (41) | 16·2 (37) | 16·3 (37) |
| FS6 cells | Normal spleen cells | 12·8 (38) | 17·0 (44) | 9·2 (41) |
| FS6 cells | — | 8·1 (32) | — | — |
| Normal C57BL mice | — | 7·5 (29) | — | — |
| Normal C57BL mice | FS6 cells | — | 9·7 (52) | — |

* % of peritoneal macrophages per exudate.

not respond to these stumuli. Over a period of 21 days after i.p. challenge, it was seen that the total number of cells peaked around Days 5 to 7, but was still somewhat elevated on Day 21.

Fig. 1 (a and b) summarizes representative experimental data on macrophage-mediated cytotoxicity for selected times over a 12-day period. Fig 1(a) shows growth inhibition and lysis induced by macrophage monolayers prepared from exudates from mice immunized and challenged with FS6 cells. It is seen that cytotoxicity was mainly non-specific. Both FS6 and FS1 cells were inhibited in their growth or were lysed, although not infrequently it was more potent towards the specific target cells. By Day 7 after challenge of mice the cytotoxicity displayed by the adherent exudate cells was occasionally specific. At this time strong growth inhibition and lysis was exerted against FS6 cells, whereas against FS1 cells there was only weak growth inhibition.

Fig 1(b) shows the cytotoxicity of macrophages from mice immunized and challenged with FS1 cells. A similar type of reaction was found, except that macrophage monolayers were almost invariably non-specifically cytotoxic. Peritoneal macrophages from unchallenged, immunized mice, or from immunized mice challenged with the non-cross-reacting tumours, or thioglycollate medium, were neither growth inhibitory nor lytic. Both anti-FS1 and anti-FS6 macrophages were also cytotoxic towards two other sygeneic fibrosarcoma cell lines, 2 allogeneic fibrosarcoma cell lines and one allogeneic lymphoma cell line (data not given).

FIG. 1(a).—Growth inhibition and lysis of FS6 cells (●——●) and FS1 cells (○——○) by peritoneal macrophages from mice immunized against and challenged i.p. with $10^6$ FS6 cells.

TABLE III.—*Cytotoxicity of peritoneal lymphocytes from immunized mice**

| Lymphocytes from | Anti-$\theta$+C′ | Lytic Index v FS6 cells | Lytic Index v FS1 cells |
|---|---|---|---|
| Mice immunized with FS6 cells | — | $25\pm5$ | 0 |
| | + | 0 | 0 |
| Mice immunized with FS1 cells | — | 0 | $21\pm3$ |
| | + | 0 | $3\pm2$ |

* Peritoneal lymphocytes harvested 6 days after specific i.p. challenge of immunized mice, were incubated for 48 h with $^{125}$IUdR-labelled tumour cells at an effector: target cell ratio of 20:1.

TABLE IV.—*Specificity of tumour-cell rejection by immunized mice after 2 i.p. challenges*

| Type of C57BL mouse | Challenged i.p. with | Rechallenged i.p. with* | No. survivors†/ No. mice challenged | Minimum survival time (days)‡ |
|---|---|---|---|---|
| Normal | — | $10^6$ FS1 | 0/5 | 16 |
| Anti-FS6 | $10^6$ FS6 | $10^6$ FS6 | 5/5 | >100 |
| Anti-FS6 | $10^6$ FS6 | $10^6$ FS1 | 0/5 | 13 |
| Anti-FS6 | $10^6$ FS6 | $10^4$ FS1 | 0/5 | 27 |
| Normal | — | $10^6$ FS6 | 0/5 | 22 |
| Normal | — | $10^4$ FS6 | 0/5 | 36 |
| Anti-FS1 | $10^6$ FS1 | $10^6$ FS1 | 5/5 | >100 |
| Anti-FS1 | $10^6$ FS1 | $10^6$ FS6 | 0/5 | 19 |
| Anti-FS1 | $10^6$ FS1 | $10^4$ FS6 | 0/5 | 40 |

* There was a 4-day interval between the 1st and 2nd i.p. challenge.
† Mice still free of tumour 90 days after challenge.
‡ Time taken for the first mouse in each group to die, after which mice that developed obvious i.p. tumours were killed to minimize trauma.

(b) *Lymphocyte-mediated cytotoxicity.*—Non-adherent peritoneal cells from immunized or normal mice were tested for their ability to lyse $^{124}$I-labelled tumour cells in a 48 h assay at an effector: target cell ratio of 201. Table III shows that in all cases lysis was specific for the immunizing antigen. The reaction was T-lymphocyte mediated, as shown by loss of lytic potential after pretreatment of cells with anti-$\theta$ serum and complement.

*Specificity of* in vivo *rejection after specific i.p. stimulation*

To ascertain whether non-specific *in vitro* cytotoxicity was a reflection of non-specific *in vivo* rejection, immunized mice were challenged i.p. with $10^6$ cells from the appropriate fibrosarcoma, to induce the appearance of cytotoxic macrophages, and then challenged i.p. with $10^4$, $10^5$ or $10^6$ cells from the non-cross-reacting fibrosarcoma at the same time or 1–5 days later. Because the results were similar throughout, only the data from Day 5 are summarized in Table IV. The minimal survival times rather than median survival times are given, because as soon as the mice in any one group began to die the remaining mice were killed to minimize trauma caused by the enlarging tumour masses and haemorrhage. The results indicated that rejection was specific in all instances. Even when threshold doses of $10^4$ unrelated cells were given, there was no difference in the survival rate compared with that of control mice.

*Adoptive transfer of immunity with peritoneal-exudate cells from tumour immunized mice*

Because of the problems associated with visualisation of the emergence and growth of i.p. tumours, Winn tests were carried out. Exudates from immunized mice challenged 5 days previously with the appropriate tumour cells ($10^6$ cells)

FIG. 1(b).—Growth inhibition and lysis of FS1 cells (●——●) and FS6 cells (○——○) by peritoneal macrophages from mice immunized against and challenged i.p. with $10^6$ FS1 cells.

or from normal mice injected with thioglycollate medium, were mixed with $10^5$ tumour cells, at a ratio of 50 peritoneal cells to 1 tumour cell, and injected i.m., as described in Materials and Methods. Growth was assessed by measuring tumour diameter. Fig. 2 (a and b) shows that immune PEC conferred protection on normal mice towards the specific immunizing tumour cells but there was no significant decrease in either the rate of emergence of unrelated tumours or in their rate of progression.

### DISCUSSION

The purpose of the above study was two-fold: firstly, to find the best method for elicitation of cytotoxic cells in tumour-immunized mice; and secondly, to attempt to correlate *in vitro* anti-tumour activity with specific rejection seen in the immunized mice (See Table I). It was found that elicitation of cytotoxicity was tumour-specific, in that injection of the unrelated tumour cells did not result in the appearance of cytotoxic macrophages or lymphocytes, even though inflammation was induced. Apart from demonstrating that the specific antigen was required to elicit cytotoxic cells, the experiments showed that the non-specific induction of an inflammatory response did not draw cytotoxic cells into the peritoneal cavity, *i.e.* induction of cytotoxicity was a localized event. Two cytotoxic cell types, macrophages and T lymphocytes, were identified. Both are regarded as essential components in both syngeneic tumour rejection and allograft rejection (Cerotinni and Brunner, 1974) whether alone or in combination with humoral factors, the macrophages perhaps amplifying T-cell cytotoxicity (Lohmann-Mathes, 1976). However, *in vitro* data indicated that while T-cell killing was always immunologically specific, macrophage cytotoxicity was in general non-specific. Specifically cytotoxic T cells have also been demonstrated in the peritoneal cavity during BCG-induced rejection of FS6 or FS1 (Parr *et al.*, 1977). As indicated previously (Evans & Alexander, 1972*a*) macrophages recovered from the peritoneal cavity of lymphoma-immunized syngeneic mice soon after i.p. challenge were non-specifically cytotoxic, but this disappeared in time and, thereafter, macrophage cytoxicity was specific for the tumour cells used for immunization. In the case of the FS6 and FS1 fibrosarcomas, the kinetics of production of cytotoxic macrophages were somewhat different from the lymphoma situation, in that only occasionally were specifically cytotoxic macrophages seen, and in

FIG. 2.—Adoptive transfer of immunity with PEC from immunized mice. (a) Mice challenged i.m. with FS6 cells + thioglycollate PEC (●——●); + anti-FS6 PEC (★——★); + anti-FS1 PEC(○——○). (b) Mice challenged i.m. with FS1 cells + thioglycollate PEC (●——●): + anti-FS1 PEC (★——★); + anti-FS6 PEC (○——○).

general cytotoxic macrophages could not be detected beyond Day 9, compared with 21–28 days in the case of lymphoma-immunized mice (Evans & Alexander, 1972*b*). The reason for this discrepancy probably lies in the nature of the tumour cells. The recognition mechanism involved in this form of non-specific macrophage-mediated cytotoxicity is not known. Although immunogenicity studies of these fibrosarcomas showed that *in vivo* rejection was wholly specific (Table I) it is possible that cytotoxic macrophages were recognising some kind of shared cross-reacting tumour-associated antigens *in vitro*, as has been shown in certain other tumour systems (Baldwin & Embeleton, 1974; Fritze *et al.*, 1975).

Despite the fact that the peritoneal cavity was apparently full of non-specifically cytotoxic macrophages for several days after specific i.p. challenge of tumour-immunized mice, we found no evidence for an innocent-bystander effect *in vivo*, in that antigenically distinct tumour cells, even in very low numbers, were not rejected at these times (Table IV). Nor was there any significant growth inhibition of unrelated tumour cells when immune peritoneal macrophages were used in Winn tests (Fig. 2). These results agree with other reports which also showed no evidence for such an effect (Klein & Klein, 1954; Zbar *et al.*, 1970). The latter report demonstrated that a guinea-pig hepatoma (Line 7) induced specific immunity after its excision, and failed to protect against an unrelated tumour, the Line 1 hepatoma. Our findings would, however, appear to differ from the several reports, summarized in an excellent review by Hibbs (1976), in which there is good reason to suppose that under conditions of adjuvant, bacterial or parasitic stimulation, non-specific macrophage cytotoxicity may be relevant to anti-tumour resistance *in vivo*. It is possible that, under these circumstances, macrophage cytotoxicity is maintained for a much longer time *in vivo*, and is therefore more effective in controlling tumour growth. Other possible reasons for the failure to demonstrate non-specific resistance in our system are (1) that the cytotoxicity was blocked or inhibited in some way and was unable to express itself *in vivo*, and (2) that perhaps the *in vitro* cytotoxic effects were exaggerated because of the prevalent cultural conditions. This second possibility, if not correct, is intrinsically of interest in that it raises an issue, muted previously, concerning the relationship between growth inhibition and lysis: whether they are reactions with similar or different pathways (Evans & Alexander, 1976). Macrophage cytotoxicity was assessed by growth inhibition and lytic assays, because of the overall evidence that macrophages may express a spectrum of cytotoxic reactivity from transient growth inhibition to irreversible lysis. In the event, this approach was justified because, when lysis was detectable, growth inhibition was unfailingly very strong, but the reverse was not necessarily true. The crux of the problem seems to lie in the importance of macrophage-mediated growth inhibition, whether under some conditions it leads to lysis directly, or whether its purpose is to predispose the cells to attack by some other effector mechanism, such as lysis by T lymphocytes.

The conclusion reached from these results is that specific rejection of FS1 and FS6 fibrosarcomas was mediated by T lymphocytes, and possibly assisted by macrophages. We found no evidence that non-specific macrophage cytotoxicity was operational *in vivo*, as measured by failure of immunized mice to reject the unrelated tumour cells, even though *in vitro* macrophages were potently cytotoxic. The suggestion is that, while non-specific macrophage cytotoxicity may play a role in surveillance by inhibiting the emergence of neoplasms, it may fail to exert an effect against tumour cells which are already relatively fast dividing.

These investigations were supported by a project grant from the Medical Research Council.

### REFERENCES

ALEXANDER, P. & EVANS, R. (1971) Endotoxin and double-stranded RNA render macrophages cytotoxic. *Nature* (*New Biol.*), **232**, 76.

BALDWIN, R. W. & EMBLETON, M. J. (1974) Neoantigens on spontaneous and carcinogen-induced rat tumours defined by *in vitro* lymphocytotoxicity assays. *Int. J. Cancer*, **13**, 433.

CEROTTINI, J. C. & BRUNNER, K. T. (1974) Cell-mediated cytotoxicity, allograft rejection and tumour immunity. *Adv. Immunol*, **18**, 67.

EVANS, R. & ALEXANDER, P. (1972*a*) Mechanisms of immunologically specific killing of tumour cells by macrophages. *Nature*, **236**, 168.

EVANS, R. & ALEXANDER, P. (1972*b*) Role of macrophages in tumour Immunity. I. Co-operation between macrophages and lymphoid cells in syngeneic tumour immunity. *Immunology*, **23**, 615. II. Involvement of a macrophage cytophilic factor during syngeneic tumour growth inhibition. *Immunology*, **23**, 627.

EVANS, R. & ALEXANDER, P. (1976) Mechanisms of extracellular killing of nucleated mammalian cells by macrophages. In *Immuno-biology of the Macrophage*. Ed. D. S. Nelson. New York: Academic Press.

FRITZE, D., KERN, D. H., WALDMAN, S. R. & PILCH, Y. H. (1975) Serological evidence for cross reacting antigens in two carcinogen-induced murine sarcomas. *Int. J. Cancer*, **15**, 116.

GHAFFER, A., CULLEN, R. T. & WOODRUFF, M. F. A. (1975) Further analysis of the anti-tumour effect *in vitro* of peritoneal exudate cells from mice treated with *Corynebacterium parvum*. *Br. J. Cancer*, **31**, 15.

HIBBS, J. B. (1974) Discrimination between neoplastic and non-neoplastic cells *in vitro* by activated macrophages. *J. Natl Cancer Inst.*, **53**, 1487.

HIBBS, J. B. (1976) The macrophage as a tumouricidal effector cell: a review of *in vivo* and *in vitro* studies on the mechanism of the activated macrophage non-specific cytotoxic reaction, In *The Macrophage in Neoplasia*. Ed. M. Fink. New York: Academic Press Inc.

KAPLAN, A. M., MORAHAN, P. S. & REGELSON, W. (1974) Induction of macrophage-mediated tumour cell cytotoxicity by pyran copolymer. *J. Natl Cancer Inst.*, **52**, 1919.

KLEIN, E. & KLEIN, G. (1954) Differential survival of solid tumour cells after inoculation into established ascites tumours. *Cancer Res.*, **14**, 139.

LOHMANN-MATHES, M. L. (1976) Induction of macrophage-mediated cytotoxicity. In *Immunobiology of the Macrophage*. Ed. D. S. Nelson. New York: Academic Press Inc.

PARR, I. B., WHEELER, E. & ALEXANDER, P. (1977) Selective mobilization of specifically cytotoxic T-lymphocytes at sites of inflammation in relation to B.G.G.-induced resistance to implants of syngeneic sarcoma in mice. *J. Natl Cancer Inst.*, **59**, 1658.

PIMM, M. V. & BALDWIN, R. W. (1975) B.C.G. immunotherapy of rat tumours in athymic nude mice. *Nature*, **254**, 77.

ZBAR, B., WEPSIC, T. H., BORSOS, T. & RAPP, H. J. (1970) Tumour-graft rejection in syngeneic guinea pigs: evidence for a two-step mechanism. *J. Natl Cancer Inst.*, **44**, 473.